# 7-Benzyl-2-[(cyclo­propyl­meth­yl)amino]-3-phenyl-5,6,7,8-tetra­hydro­pyrido[4′,3′:4,5]thieno[2,3-*d*]pyrimidin-4(3*H*)-one

**DOI:** 10.1107/S1600536811029771

**Published:** 2011-07-30

**Authors:** Hong Chen

**Affiliations:** a College of Chemistry and Life Science, China Three Gorges University, Yichang 443002, People’s Republic of China

## Abstract

There are two independent mol­ecules in the asymmetric unit of the title compound, C_26_H_26_N_4_OS. In each mol­ecule, the thienopyrimidine fused-ring system is essentially planar with a maximum deviation of 0.0409 (18) for the N atom. In one mol­ecule, this ring system forms diherdral angles of 84.8 (1) and 67.6 (1)° with the adjacent phenyl and benzyl rings, respectively, while the corresponding angles in the other mol­ecule are 77.9 (1) and 66.5 (1)°.

## Related literature

For the biological activity of thienopyrimidine-containing compounds, see: Amr *et al.* (2010 ▶); Huang *et al.* (2009 ▶); Jennings *et al.* (2005 ▶); Kikuchi *et al.* (2006 ▶); Mavrova *et al.* (2010 ▶); Santagati *et al.* (2002 ▶). For related structures, see: Xie *et al.* (2008 ▶); Hu *et al.* (2007 ▶).
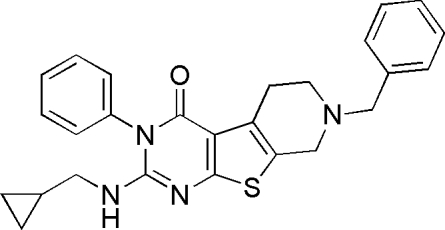


## Experimental

### 

#### Crystal data

C_26_H_26_N_4_OS
*M* *_r_* = 442.58
Orthorhombic, 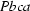

*a* = 18.269 (5) Å
*b* = 27.564 (7) Å
*c* = 18.115 (5) Å
*V* = 9122 (4) Å^3^
*Z* = 16
Mo *K*α radiation
μ = 0.17 mm^−1^
*T* = 296 K
0.23 × 0.19 × 0.15 mm

#### Data collection

Bruker SMART CCD diffractometer
Absorption correction: multi-scan (*SADABS*; Sheldrick, 1996 ▶) *T*_min_ = 0.962, *T*_max_ = 0.975
92541 measured reflections
10442 independent reflections
7717 reflections with *I* > 2σ(*I*)
*R*_int_ = 0.067

#### Refinement

*R*[*F* ^2^ > 2σ(*F* ^2^)] = 0.058
*wR*(*F* ^2^) = 0.157
*S* = 1.06
10442 reflections
577 parameters
H-atom parameters constrained
Δρ_max_ = 0.39 e Å^−3^
Δρ_min_ = −0.28 e Å^−3^

### 

Data collection: *SMART* (Bruker, 1997 ▶); cell refinement: *SAINT* (Bruker, 1997 ▶); data reduction: *SAINT*; program(s) used to solve structure: *SHELXTL* (Sheldrick, 2008 ▶); program(s) used to refine structure: *SHELXTL*; molecular graphics: *SHELXTL*; software used to prepare material for publication: *SHELXTL*.

## Supplementary Material

Crystal structure: contains datablock(s) I, global. DOI: 10.1107/S1600536811029771/lh5288sup1.cif

Structure factors: contains datablock(s) I. DOI: 10.1107/S1600536811029771/lh5288Isup2.hkl

Supplementary material file. DOI: 10.1107/S1600536811029771/lh5288Isup3.cml

Additional supplementary materials:  crystallographic information; 3D view; checkCIF report

## Figures and Tables

**Fig. 1.**
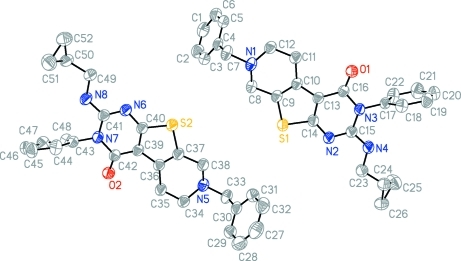
The molecular structure of the title compound with 50% probability displacement ellipsoids.

**Fig. 2.**
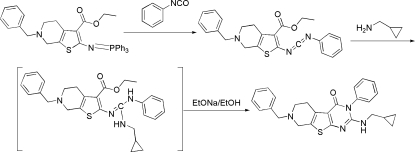
The reaction scheme.

## supplementary crystallographic information

### Comment

Derivatives of heterocycles containing the thienopyrimidine system have proved to show significant antifungal, antibacterical, anticonvulsant and angiotensin antagonistic activities (Amr *et al.* 2010; Huang *et al.* 2009; Jennings *et al.* 2005; Kikuchi *et al.* 2006; Mavrova *et al.* 2010; Santagati *et al.* 2002). Recently, we have focused on the synthesis of fused heterocyclic systems containing thienopyrimidine *via* aza-wittig reactions under mild conditions. Some examples of crystal structures of fused pyrimidinone derivatives have been reported (Xie *et al.*, 2008; Hu *et al.*, 2007). The title compound (I) has potential use as a precursor for obtaining bioactive molecules with fluorescence properties. Herein, we report the crystal structure of (I).

The asymmetric unit of the title compound is shown in Fig. 1. There are two crystallographically independent molecules per asymmetric unit. In each, the thienopyrimidine fused ring system is essentially planar. In one molecule this ring system forms diherdral angles of 84.8 (1) and 67.6 (1) ° with the adjacent phenyl (C17-22) ring and benzyl phenyl ring (C1-C6), respectively, and the corresponding angles in the other molecule are 77.9 (1)° for C43-C48 and 66.5 (1)° for C28-C32.

### Experimental

A schematic of the synthesis is given in Fig. 2. 1-Isocyanatobenzene (2 mmol) under nitrogen atmosphere was added to a solution of iminophosphorane (2 mmol) in anhydrous CH_2_Cl_2_ (10 ml) at room temperature. When the reaction mixture was left unstirred for 12 h at 273-278K, iminophosphorane was added (TLC monitored). The solvent was removed under reduced pressure and ether/petroleum ether (volume ratio 1:2, 20 ml) was added to precipitate triphenylphosphine oxide. Removal of the solvent gave the carbodiimide, which was used directly without further purification. Cyclopropylmethanamine (2 mmol) was added to the solution of carbodiimide in anhydrous dichloromethane (10 ml). After the reaction mixture was left unstirred for 5–6 h, the solvent was removed and anhydrous EtOH (10 ml) with several drops of EtONa (in EtOH) was added to the mixture. The mixture was stirred for another 6–8 h at room temperature. The solution was condensed and the residual was recrystallized from EtOH to give the expected title compound as white crystals.

### Refinement

All H atoms were positioned geometrically [C—H = 0.93, 0.97 Å and N—H = 0.86Å] and allowed to ride on their parent atoms, with *U*_iso_(H)=1.2*U*_eq_(C, N).

### Crystal data

**Table:**

C_26_H_26_N_4_OS	*D*_x_ = 1.289 Mg m^−^^3^
*M**_r_* = 442.58	Melting point: 469 K
Orthorhombic, *P**b**c**a*	Mo *K*α radiation, λ = 0.71073 Å
Hall symbol: -P 2ac 2ab	Cell parameters from 2256 reflections
*a* = 18.269 (5) Å	θ = 1.8–27.5°
*b* = 27.564 (7) Å	µ = 0.17 mm^−^^1^
*c* = 18.115 (5) Å	*T* = 296 K
*V* = 9122 (4) Å^3^	Block, colourless
*Z* = 16	0.23 × 0.19 × 0.15 mm
*F*(000) = 3744	

### Data collection

**Table:**

Bruker SMART CCD diffractometer	10442 independent reflections
Radiation source: fine-focus sealed tube	7717 reflections with *I* > 2σ(*I*)
graphite	*R*_int_ = 0.067
CCD Profile fitting scans	θ_max_ = 27.5°, θ_min_ = 1.8°
Absorption correction: multi-scan (*SADABS*; Sheldrick, 1996)	*h* = −23→23
*T*_min_ = 0.962, *T*_max_ = 0.975	*k* = −35→35
92541 measured reflections	*l* = −23→23

### Refinement

**Table:**

Refinement on *F*^2^	Primary atom site location: structure-invariant direct methods
Least-squares matrix: full	Secondary atom site location: difference Fourier map
*R*[*F*^2^ > 2σ(*F*^2^)] = 0.058	Hydrogen site location: inferred from neighbouring sites
*wR*(*F*^2^) = 0.157	H-atom parameters constrained
*S* = 1.06	*w* = 1/[σ^2^(*F*_o_^2^) + (0.0619*P*)^2^ + 4.0594*P*] where *P* = (*F*_o_^2^ + 2*F*_c_^2^)/3
10442 reflections	(Δ/σ)_max_ = 0.001
577 parameters	Δρ_max_ = 0.39 e Å^−^^3^
0 restraints	Δρ_min_ = −0.28 e Å^−^^3^

### Special details

**Table:**

Geometry. All e.s.d.'s (except the e.s.d. in the dihedral angle between two l.s. planes) are estimated using the full covariance matrix. The cell e.s.d.'s are taken into account individually in the estimation of e.s.d.'s in distances, angles and torsion angles; correlations between e.s.d.'s in cell parameters are only used when they are defined by crystal symmetry. An approximate (isotropic) treatment of cell e.s.d.'s is used for estimating e.s.d.'s involving l.s. planes.
Refinement. Refinement of *F*^2^ against ALL reflections. The weighted *R*-factor *wR* and goodness of fit *S* are based on *F*^2^, conventional *R*-factors *R* are based on *F*, with *F* set to zero for negative *F*^2^. The threshold expression of *F*^2^ > σ(*F*^2^) is used only for calculating *R*-factors(gt) *etc*. and is not relevant to the choice of reflections for refinement. *R*-factors based on *F*^2^ are statistically about twice as large as those based on *F*, and *R*- factors based on ALL data will be even larger.

### Fractional atomic coordinates and isotropic or equivalent isotropic displacement parameters (Å^2^)

**Table:**

	*x*	*y*	*z*	*U*_iso_*/*U*_eq_	
C1	0.05171 (15)	0.18192 (10)	−0.00244 (14)	0.0660 (7)	
H1A	0.0104	0.1913	−0.0289	0.079*	
C2	0.09443 (16)	0.21617 (10)	0.03172 (14)	0.0652 (7)	
H2B	0.0821	0.2488	0.0284	0.078*	
C3	0.15569 (14)	0.20216 (8)	0.07097 (13)	0.0554 (6)	
H3A	0.1848	0.2257	0.0931	0.066*	
C4	0.17479 (12)	0.15340 (8)	0.07805 (11)	0.0464 (5)	
C5	0.13115 (14)	0.11913 (8)	0.04293 (12)	0.0540 (6)	
H5A	0.1428	0.0864	0.0465	0.065*	
C6	0.07028 (15)	0.13342 (10)	0.00261 (14)	0.0633 (6)	
H6B	0.0418	0.1102	−0.0212	0.076*	
C7	0.24058 (13)	0.13822 (9)	0.12318 (12)	0.0541 (6)	
H7A	0.2626	0.1102	0.0996	0.065*	
H7B	0.2762	0.1643	0.1216	0.065*	
C8	0.20203 (13)	0.16794 (8)	0.24430 (11)	0.0477 (5)	
H8B	0.1580	0.1814	0.2226	0.057*	
H8C	0.2396	0.1928	0.2440	0.057*	
C9	0.18688 (11)	0.15206 (8)	0.32244 (11)	0.0435 (5)	
C10	0.17911 (11)	0.10554 (7)	0.34433 (11)	0.0415 (4)	
C11	0.18447 (13)	0.06478 (8)	0.28952 (11)	0.0473 (5)	
H11A	0.1460	0.0413	0.2989	0.057*	
H11B	0.2312	0.0485	0.2949	0.057*	
C12	0.17709 (12)	0.08452 (8)	0.21114 (12)	0.0483 (5)	
H12A	0.1893	0.0593	0.1758	0.058*	
H12B	0.1270	0.0946	0.2024	0.058*	
C13	0.16484 (11)	0.10186 (7)	0.42242 (11)	0.0406 (4)	
C14	0.16229 (12)	0.14674 (7)	0.45695 (11)	0.0443 (5)	
C15	0.13456 (13)	0.11724 (7)	0.56918 (12)	0.0479 (5)	
C16	0.15004 (11)	0.05948 (7)	0.46609 (11)	0.0406 (4)	
C17	0.11002 (11)	0.03031 (7)	0.58726 (10)	0.0396 (4)	
C18	0.16081 (12)	0.00189 (8)	0.62358 (12)	0.0484 (5)	
H18A	0.2106	0.0073	0.6170	0.058*	
C19	0.13676 (14)	−0.03480 (9)	0.66992 (13)	0.0563 (6)	
H19A	0.1706	−0.0541	0.6946	0.068*	
C20	0.06353 (14)	−0.04282 (9)	0.67949 (13)	0.0588 (6)	
H20A	0.0477	−0.0672	0.7113	0.071*	
C21	0.01325 (14)	−0.01502 (10)	0.64232 (15)	0.0667 (7)	
H21A	−0.0365	−0.0210	0.6485	0.080*	
C22	0.03600 (12)	0.02173 (9)	0.59576 (13)	0.0558 (6)	
H22A	0.0019	0.0405	0.5705	0.067*	
C23	0.12097 (17)	0.16989 (9)	0.67740 (13)	0.0646 (7)	
H23A	0.0831	0.1905	0.6568	0.078*	
H23B	0.1679	0.1853	0.6687	0.078*	
C24	0.10890 (15)	0.16393 (9)	0.75867 (14)	0.0605 (6)	
H24A	0.1433	0.1426	0.7846	0.073*	
C25	0.03379 (18)	0.16520 (10)	0.78776 (19)	0.0812 (9)	
H25A	0.0225	0.1446	0.8297	0.097*	
H25B	−0.0059	0.1691	0.7527	0.097*	
C26	0.08291 (18)	0.20727 (10)	0.80036 (16)	0.0754 (8)	
H26A	0.1018	0.2122	0.8498	0.090*	
H26B	0.0734	0.2368	0.7727	0.090*	
C27	0.45806 (16)	0.31675 (11)	0.53400 (15)	0.0712 (7)	
H27A	0.4981	0.3051	0.5602	0.085*	
C28	0.44822 (15)	0.36614 (11)	0.52499 (15)	0.0677 (7)	
H28A	0.4815	0.3878	0.5455	0.081*	
C29	0.38873 (14)	0.38345 (9)	0.48537 (13)	0.0554 (6)	
H29A	0.3822	0.4167	0.4799	0.066*	
C30	0.33877 (12)	0.35166 (8)	0.45376 (11)	0.0460 (5)	
C31	0.34932 (14)	0.30222 (9)	0.46439 (13)	0.0568 (6)	
H31A	0.3159	0.2804	0.4444	0.068*	
C32	0.40810 (17)	0.28488 (10)	0.50385 (15)	0.0695 (7)	
H32A	0.4142	0.2516	0.5102	0.083*	
C33	0.27431 (12)	0.37010 (9)	0.40907 (11)	0.0523 (5)	
H33A	0.2354	0.3462	0.4120	0.063*	
H33B	0.2564	0.3996	0.4322	0.063*	
C34	0.33811 (13)	0.42117 (8)	0.31735 (12)	0.0500 (5)	
H34A	0.3882	0.4107	0.3247	0.060*	
H34B	0.3278	0.4470	0.3522	0.060*	
C35	0.32851 (13)	0.43987 (8)	0.23847 (12)	0.0500 (5)	
H35A	0.2823	0.4570	0.2343	0.060*	
H35B	0.3676	0.4624	0.2267	0.060*	
C36	0.32975 (11)	0.39816 (7)	0.18526 (11)	0.0436 (5)	
C37	0.32282 (12)	0.35191 (8)	0.20944 (12)	0.0462 (5)	
C38	0.31096 (13)	0.33769 (8)	0.28888 (12)	0.0493 (5)	
H38A	0.2735	0.3128	0.2920	0.059*	
H38B	0.3559	0.3248	0.3095	0.059*	
C39	0.34055 (11)	0.40028 (7)	0.10637 (11)	0.0424 (4)	
C40	0.34265 (12)	0.35469 (7)	0.07409 (11)	0.0438 (5)	
C41	0.36639 (12)	0.38217 (7)	−0.04050 (11)	0.0444 (5)	
C42	0.35082 (11)	0.44197 (7)	0.05997 (11)	0.0414 (4)	
C43	0.38726 (11)	0.46859 (7)	−0.06462 (11)	0.0412 (4)	
C44	0.45998 (12)	0.47448 (9)	−0.08320 (13)	0.0547 (6)	
H44A	0.4956	0.4551	−0.0614	0.066*	
C45	0.47957 (13)	0.50931 (10)	−0.13446 (14)	0.0611 (6)	
H45A	0.5284	0.5131	−0.1477	0.073*	
C46	0.42712 (14)	0.53828 (8)	−0.16571 (13)	0.0569 (6)	
H46A	0.4404	0.5614	−0.2006	0.068*	
C47	0.35511 (14)	0.53343 (8)	−0.14577 (13)	0.0558 (6)	
H47A	0.3200	0.5538	−0.1663	0.067*	
C48	0.33424 (12)	0.49826 (8)	−0.09518 (12)	0.0476 (5)	
H48A	0.2853	0.4947	−0.0820	0.057*	
C49	0.38305 (14)	0.32679 (8)	−0.14454 (12)	0.0529 (5)	
H49A	0.3355	0.3114	−0.1404	0.063*	
H49B	0.4182	0.3073	−0.1176	0.063*	
C50	0.40478 (14)	0.32943 (9)	−0.22364 (13)	0.0545 (6)	
H50A	0.3729	0.3485	−0.2560	0.065*	
C51	0.48394 (17)	0.32917 (10)	−0.24251 (19)	0.0797 (8)	
H51A	0.4996	0.3481	−0.2848	0.096*	
H51B	0.5188	0.3279	−0.2022	0.096*	
C52	0.4386 (2)	0.28530 (10)	−0.25664 (18)	0.0881 (10)	
H52A	0.4459	0.2574	−0.2248	0.106*	
H52B	0.4267	0.2776	−0.3075	0.106*	
N1	0.22645 (9)	0.12606 (7)	0.20132 (9)	0.0465 (4)	
N2	0.14885 (11)	0.15593 (6)	0.52925 (10)	0.0501 (4)	
N3	0.13345 (10)	0.07020 (6)	0.54068 (9)	0.0439 (4)	
N4	0.11928 (13)	0.12239 (7)	0.64157 (10)	0.0621 (6)	
H4A	0.1083	0.0972	0.6672	0.075*	
N5	0.28822 (10)	0.38053 (6)	0.33019 (10)	0.0468 (4)	
N6	0.35401 (11)	0.34421 (6)	0.00171 (9)	0.0481 (4)	
N7	0.36687 (10)	0.42972 (6)	−0.01448 (9)	0.0434 (4)	
N8	0.38000 (11)	0.37522 (7)	−0.11277 (10)	0.0536 (5)	
H8A	0.3870	0.4000	−0.1408	0.064*	
O1	0.15003 (9)	0.01718 (5)	0.44508 (8)	0.0497 (4)	
O2	0.34838 (9)	0.48479 (5)	0.07846 (8)	0.0506 (4)	
S1	0.17720 (4)	0.193556 (19)	0.39458 (3)	0.05154 (16)	
S2	0.33002 (4)	0.30915 (2)	0.13895 (3)	0.05282 (16)	

### Atomic displacement parameters (Å^2^)

**Table:**

	*U*^11^	*U*^22^	*U*^33^	*U*^12^	*U*^13^	*U*^23^
C1	0.0654 (16)	0.0817 (18)	0.0507 (14)	0.0074 (14)	−0.0009 (12)	0.0130 (13)
C2	0.0839 (19)	0.0574 (14)	0.0542 (15)	0.0118 (13)	−0.0006 (13)	0.0080 (12)
C3	0.0708 (15)	0.0513 (12)	0.0441 (12)	−0.0057 (11)	0.0020 (11)	0.0025 (10)
C4	0.0525 (12)	0.0534 (12)	0.0331 (10)	0.0016 (10)	0.0112 (9)	0.0048 (9)
C5	0.0706 (15)	0.0503 (12)	0.0411 (12)	−0.0016 (11)	0.0066 (11)	0.0035 (9)
C6	0.0681 (16)	0.0726 (16)	0.0493 (14)	−0.0156 (13)	0.0022 (12)	0.0033 (12)
C7	0.0507 (13)	0.0698 (14)	0.0417 (12)	0.0062 (11)	0.0131 (10)	0.0079 (10)
C8	0.0511 (12)	0.0530 (12)	0.0390 (11)	−0.0004 (10)	0.0055 (9)	0.0058 (9)
C9	0.0458 (11)	0.0478 (11)	0.0368 (10)	0.0030 (9)	0.0049 (8)	0.0015 (9)
C10	0.0430 (10)	0.0457 (11)	0.0359 (10)	0.0029 (9)	0.0047 (8)	0.0014 (8)
C11	0.0536 (12)	0.0473 (11)	0.0410 (11)	0.0021 (10)	0.0086 (9)	−0.0010 (9)
C12	0.0517 (12)	0.0534 (12)	0.0397 (11)	0.0045 (10)	0.0069 (9)	−0.0017 (9)
C13	0.0432 (10)	0.0425 (10)	0.0362 (10)	0.0037 (8)	0.0033 (8)	0.0001 (8)
C14	0.0485 (11)	0.0433 (11)	0.0410 (11)	0.0009 (9)	0.0052 (9)	0.0022 (8)
C15	0.0610 (13)	0.0432 (11)	0.0395 (11)	0.0022 (10)	0.0080 (10)	−0.0015 (9)
C16	0.0395 (10)	0.0454 (11)	0.0368 (10)	0.0036 (9)	0.0037 (8)	0.0003 (8)
C17	0.0461 (11)	0.0392 (10)	0.0336 (10)	0.0021 (8)	0.0050 (8)	0.0005 (8)
C18	0.0416 (11)	0.0536 (12)	0.0501 (12)	0.0030 (10)	−0.0010 (9)	0.0040 (10)
C19	0.0627 (15)	0.0547 (13)	0.0515 (13)	0.0110 (11)	−0.0040 (11)	0.0121 (11)
C20	0.0713 (16)	0.0521 (13)	0.0529 (14)	−0.0102 (12)	0.0048 (12)	0.0098 (11)
C21	0.0480 (13)	0.0821 (18)	0.0701 (17)	−0.0141 (13)	0.0058 (12)	0.0166 (14)
C22	0.0411 (11)	0.0700 (15)	0.0564 (14)	0.0058 (11)	0.0002 (10)	0.0147 (12)
C23	0.097 (2)	0.0479 (12)	0.0487 (13)	−0.0058 (13)	0.0159 (13)	−0.0049 (10)
C24	0.0746 (17)	0.0510 (13)	0.0560 (14)	0.0036 (12)	0.0043 (12)	−0.0059 (11)
C25	0.087 (2)	0.0645 (17)	0.092 (2)	0.0020 (15)	0.0228 (17)	−0.0099 (15)
C26	0.112 (2)	0.0563 (15)	0.0576 (16)	0.0002 (15)	0.0072 (15)	−0.0163 (12)
C27	0.0664 (17)	0.089 (2)	0.0582 (16)	0.0180 (15)	−0.0012 (13)	0.0089 (14)
C28	0.0642 (16)	0.0819 (19)	0.0570 (15)	−0.0086 (14)	−0.0004 (12)	−0.0063 (13)
C29	0.0653 (15)	0.0512 (13)	0.0497 (13)	−0.0017 (11)	0.0096 (11)	−0.0010 (10)
C30	0.0524 (12)	0.0492 (11)	0.0364 (10)	0.0021 (10)	0.0112 (9)	0.0012 (9)
C31	0.0690 (15)	0.0496 (12)	0.0518 (13)	−0.0025 (11)	0.0037 (11)	0.0010 (10)
C32	0.088 (2)	0.0590 (15)	0.0611 (16)	0.0146 (14)	0.0052 (14)	0.0078 (12)
C33	0.0514 (12)	0.0661 (14)	0.0393 (11)	0.0068 (11)	0.0117 (9)	0.0028 (10)
C34	0.0586 (13)	0.0499 (12)	0.0414 (11)	0.0031 (10)	0.0055 (10)	−0.0045 (9)
C35	0.0630 (14)	0.0452 (11)	0.0418 (11)	0.0012 (10)	0.0098 (10)	−0.0012 (9)
C36	0.0468 (11)	0.0443 (11)	0.0397 (11)	0.0031 (9)	0.0058 (9)	−0.0024 (8)
C37	0.0527 (12)	0.0465 (11)	0.0395 (11)	0.0025 (9)	0.0048 (9)	−0.0011 (9)
C38	0.0594 (13)	0.0472 (11)	0.0414 (11)	0.0016 (10)	0.0058 (10)	0.0013 (9)
C39	0.0454 (11)	0.0449 (10)	0.0370 (10)	0.0047 (9)	0.0056 (8)	−0.0029 (8)
C40	0.0502 (11)	0.0429 (11)	0.0383 (11)	0.0028 (9)	0.0041 (9)	−0.0002 (8)
C41	0.0512 (12)	0.0419 (10)	0.0400 (11)	0.0047 (9)	0.0042 (9)	−0.0023 (8)
C42	0.0410 (10)	0.0438 (10)	0.0394 (10)	0.0055 (9)	0.0046 (8)	−0.0016 (8)
C43	0.0454 (11)	0.0413 (10)	0.0369 (10)	0.0036 (9)	0.0019 (8)	0.0013 (8)
C44	0.0431 (12)	0.0662 (14)	0.0547 (13)	0.0098 (11)	0.0009 (10)	0.0116 (11)
C45	0.0468 (13)	0.0769 (17)	0.0597 (15)	−0.0088 (12)	0.0066 (11)	0.0104 (13)
C46	0.0714 (16)	0.0507 (12)	0.0486 (13)	−0.0069 (12)	0.0019 (11)	0.0071 (10)
C47	0.0625 (15)	0.0492 (12)	0.0557 (14)	0.0098 (11)	−0.0043 (11)	0.0092 (10)
C48	0.0425 (11)	0.0507 (12)	0.0496 (12)	0.0055 (9)	−0.0010 (9)	0.0014 (10)
C49	0.0635 (14)	0.0501 (12)	0.0450 (12)	−0.0002 (11)	0.0066 (10)	−0.0071 (10)
C50	0.0618 (14)	0.0566 (13)	0.0450 (12)	0.0029 (11)	0.0041 (10)	−0.0096 (10)
C51	0.0760 (19)	0.0718 (17)	0.091 (2)	−0.0004 (15)	0.0305 (16)	−0.0094 (16)
C52	0.125 (3)	0.0602 (16)	0.079 (2)	−0.0033 (17)	0.0348 (19)	−0.0221 (15)
N1	0.0448 (9)	0.0573 (11)	0.0375 (9)	0.0045 (8)	0.0088 (7)	0.0042 (8)
N2	0.0704 (12)	0.0413 (9)	0.0385 (9)	0.0000 (9)	0.0092 (9)	−0.0008 (7)
N3	0.0561 (10)	0.0397 (9)	0.0358 (9)	0.0022 (8)	0.0067 (8)	0.0022 (7)
N4	0.1036 (17)	0.0432 (10)	0.0395 (10)	−0.0051 (10)	0.0185 (10)	−0.0021 (8)
N5	0.0485 (10)	0.0528 (10)	0.0391 (9)	0.0044 (8)	0.0082 (8)	0.0017 (8)
N6	0.0634 (12)	0.0427 (9)	0.0382 (9)	0.0018 (8)	0.0054 (8)	−0.0019 (7)
N7	0.0522 (10)	0.0399 (9)	0.0380 (9)	0.0049 (8)	0.0055 (7)	0.0022 (7)
N8	0.0777 (13)	0.0446 (10)	0.0385 (10)	0.0064 (9)	0.0092 (9)	−0.0015 (8)
O1	0.0640 (10)	0.0406 (8)	0.0444 (8)	0.0043 (7)	0.0078 (7)	−0.0025 (6)
O2	0.0641 (10)	0.0399 (7)	0.0479 (9)	0.0062 (7)	0.0063 (7)	−0.0039 (6)
S1	0.0699 (4)	0.0420 (3)	0.0427 (3)	0.0003 (3)	0.0081 (3)	0.0033 (2)
S2	0.0762 (4)	0.0406 (3)	0.0416 (3)	−0.0003 (3)	0.0067 (3)	−0.0004 (2)

### Geometric parameters (Å, °)

**Table:**

C1—C2	1.372 (4)	C27—C28	1.383 (4)
C1—C6	1.382 (4)	C27—H27A	0.9300
C1—H1A	0.9300	C28—C29	1.387 (4)
C2—C3	1.381 (4)	C28—H28A	0.9300
C2—H2B	0.9300	C29—C30	1.389 (3)
C3—C4	1.394 (3)	C29—H29A	0.9300
C3—H3A	0.9300	C30—C31	1.390 (3)
C4—C5	1.390 (3)	C30—C33	1.517 (3)
C4—C7	1.513 (3)	C31—C32	1.376 (4)
C5—C6	1.387 (4)	C31—H31A	0.9300
C5—H5A	0.9300	C32—H32A	0.9300
C6—H6B	0.9300	C33—N5	1.479 (3)
C7—N1	1.477 (3)	C33—H33A	0.9700
C7—H7A	0.9700	C33—H33B	0.9700
C7—H7B	0.9700	C34—N5	1.463 (3)
C8—N1	1.462 (3)	C34—C35	1.529 (3)
C8—C9	1.507 (3)	C34—H34A	0.9700
C8—H8B	0.9700	C34—H34B	0.9700
C8—H8C	0.9700	C35—C36	1.500 (3)
C9—C10	1.350 (3)	C35—H35A	0.9700
C9—S1	1.746 (2)	C35—H35B	0.9700
C10—C13	1.442 (3)	C36—C37	1.354 (3)
C10—C11	1.503 (3)	C36—C39	1.444 (3)
C11—C12	1.527 (3)	C37—C38	1.507 (3)
C11—H11A	0.9700	C37—S2	1.743 (2)
C11—H11B	0.9700	C38—N5	1.458 (3)
C12—N1	1.468 (3)	C38—H38A	0.9700
C12—H12A	0.9700	C38—H38B	0.9700
C12—H12B	0.9700	C39—C40	1.387 (3)
C13—C14	1.387 (3)	C39—C42	1.436 (3)
C13—C16	1.436 (3)	C40—N6	1.359 (3)
C14—N2	1.356 (3)	C40—S2	1.735 (2)
C14—S1	1.737 (2)	C41—N6	1.316 (3)
C15—N2	1.315 (3)	C41—N8	1.346 (3)
C15—N4	1.348 (3)	C41—N7	1.393 (3)
C15—N3	1.396 (3)	C42—O2	1.228 (2)
C16—O1	1.227 (2)	C42—N7	1.421 (3)
C16—N3	1.416 (3)	C43—C44	1.380 (3)
C17—C18	1.381 (3)	C43—C48	1.383 (3)
C17—C22	1.381 (3)	C43—N7	1.453 (2)
C17—N3	1.451 (2)	C44—C45	1.383 (3)
C18—C19	1.386 (3)	C44—H44A	0.9300
C18—H18A	0.9300	C45—C46	1.370 (3)
C19—C20	1.367 (3)	C45—H45A	0.9300
C19—H19A	0.9300	C46—C47	1.371 (3)
C20—C21	1.373 (4)	C46—H46A	0.9300
C20—H20A	0.9300	C47—C48	1.388 (3)
C21—C22	1.382 (3)	C47—H47A	0.9300
C21—H21A	0.9300	C48—H48A	0.9300
C22—H22A	0.9300	C49—N8	1.455 (3)
C23—N4	1.462 (3)	C49—C50	1.489 (3)
C23—C24	1.498 (3)	C49—H49A	0.9700
C23—H23A	0.9700	C49—H49B	0.9700
C23—H23B	0.9700	C50—C51	1.486 (4)
C24—C25	1.470 (4)	C50—C52	1.490 (3)
C24—C26	1.491 (3)	C50—H50A	0.9800
C24—H24A	0.9800	C51—C52	1.488 (4)
C25—C26	1.484 (4)	C51—H51A	0.9700
C25—H25A	0.9700	C51—H51B	0.9700
C25—H25B	0.9700	C52—H52A	0.9700
C26—H26A	0.9700	C52—H52B	0.9700
C26—H26B	0.9700	N4—H4A	0.8600
C27—C32	1.379 (4)	N8—H8A	0.8600
			
C2—C1—C6	119.7 (3)	C29—C30—C33	121.3 (2)
C2—C1—H1A	120.1	C32—C31—C30	121.4 (2)
C6—C1—H1A	120.1	C32—C31—H31A	119.3
C1—C2—C3	120.0 (2)	C30—C31—H31A	119.3
C1—C2—H2B	120.0	C31—C32—C27	120.1 (3)
C3—C2—H2B	120.0	C31—C32—H32A	120.0
C2—C3—C4	121.3 (2)	C27—C32—H32A	120.0
C2—C3—H3A	119.3	N5—C33—C30	116.57 (17)
C4—C3—H3A	119.3	N5—C33—H33A	108.2
C3—C4—C5	118.0 (2)	C30—C33—H33A	108.2
C3—C4—C7	121.0 (2)	N5—C33—H33B	108.2
C5—C4—C7	121.0 (2)	C30—C33—H33B	108.2
C6—C5—C4	120.5 (2)	H33A—C33—H33B	107.3
C6—C5—H5A	119.8	N5—C34—C35	109.59 (18)
C4—C5—H5A	119.8	N5—C34—H34A	109.8
C1—C6—C5	120.4 (2)	C35—C34—H34A	109.8
C1—C6—H6B	119.8	N5—C34—H34B	109.8
C5—C6—H6B	119.8	C35—C34—H34B	109.8
N1—C7—C4	116.22 (17)	H34A—C34—H34B	108.2
N1—C7—H7A	108.2	C36—C35—C34	109.89 (18)
C4—C7—H7A	108.2	C36—C35—H35A	109.7
N1—C7—H7B	108.2	C34—C35—H35A	109.7
C4—C7—H7B	108.2	C36—C35—H35B	109.7
H7A—C7—H7B	107.4	C34—C35—H35B	109.7
N1—C8—C9	109.07 (17)	H35A—C35—H35B	108.2
N1—C8—H8B	109.9	C37—C36—C39	111.78 (18)
C9—C8—H8B	109.9	C37—C36—C35	120.82 (19)
N1—C8—H8C	109.9	C39—C36—C35	127.36 (19)
C9—C8—H8C	109.9	C36—C37—C38	124.55 (19)
H8B—C8—H8C	108.3	C36—C37—S2	113.13 (16)
C10—C9—C8	124.83 (19)	C38—C37—S2	122.32 (16)
C10—C9—S1	113.07 (15)	N5—C38—C37	108.69 (17)
C8—C9—S1	122.09 (16)	N5—C38—H38A	110.0
C9—C10—C13	111.97 (18)	C37—C38—H38A	110.0
C9—C10—C11	120.63 (18)	N5—C38—H38B	110.0
C13—C10—C11	127.40 (18)	C37—C38—H38B	110.0
C10—C11—C12	110.01 (17)	H38A—C38—H38B	108.3
C10—C11—H11A	109.7	C40—C39—C42	118.34 (18)
C12—C11—H11A	109.7	C40—C39—C36	112.61 (18)
C10—C11—H11B	109.7	C42—C39—C36	129.02 (18)
C12—C11—H11B	109.7	N6—C40—C39	127.15 (19)
H11A—C11—H11B	108.2	N6—C40—S2	121.35 (15)
N1—C12—C11	109.67 (18)	C39—C40—S2	111.49 (15)
N1—C12—H12A	109.7	N6—C41—N8	118.93 (19)
C11—C12—H12A	109.7	N6—C41—N7	123.57 (18)
N1—C12—H12B	109.7	N8—C41—N7	117.49 (18)
C11—C12—H12B	109.7	O2—C42—N7	119.66 (18)
H12A—C12—H12B	108.2	O2—C42—C39	127.22 (19)
C14—C13—C16	118.07 (18)	N7—C42—C39	113.11 (17)
C14—C13—C10	112.69 (18)	C44—C43—C48	120.5 (2)
C16—C13—C10	129.16 (18)	C44—C43—N7	119.07 (18)
N2—C14—C13	127.46 (19)	C48—C43—N7	120.43 (19)
N2—C14—S1	121.18 (16)	C45—C44—C43	119.6 (2)
C13—C14—S1	111.35 (15)	C45—C44—H44A	120.2
N2—C15—N4	119.39 (19)	C43—C44—H44A	120.2
N2—C15—N3	123.57 (19)	C46—C45—C44	120.1 (2)
N4—C15—N3	117.04 (18)	C46—C45—H45A	120.0
O1—C16—N3	119.63 (18)	C44—C45—H45A	120.0
O1—C16—C13	127.03 (18)	C45—C46—C47	120.4 (2)
N3—C16—C13	113.34 (17)	C45—C46—H46A	119.8
C18—C17—C22	120.50 (19)	C47—C46—H46A	119.8
C18—C17—N3	120.58 (19)	C46—C47—C48	120.4 (2)
C22—C17—N3	118.91 (19)	C46—C47—H47A	119.8
C17—C18—C19	119.3 (2)	C48—C47—H47A	119.8
C17—C18—H18A	120.3	C43—C48—C47	119.0 (2)
C19—C18—H18A	120.3	C43—C48—H48A	120.5
C20—C19—C18	120.3 (2)	C47—C48—H48A	120.5
C20—C19—H19A	119.8	N8—C49—C50	110.26 (19)
C18—C19—H19A	119.8	N8—C49—H49A	109.6
C19—C20—C21	120.2 (2)	C50—C49—H49A	109.6
C19—C20—H20A	119.9	N8—C49—H49B	109.6
C21—C20—H20A	119.9	C50—C49—H49B	109.6
C20—C21—C22	120.5 (2)	H49A—C49—H49B	108.1
C20—C21—H21A	119.8	C51—C50—C52	59.99 (19)
C22—C21—H21A	119.8	C51—C50—C49	118.7 (2)
C21—C22—C17	119.2 (2)	C52—C50—C49	117.2 (2)
C21—C22—H22A	120.4	C51—C50—H50A	116.4
C17—C22—H22A	120.4	C52—C50—H50A	116.4
N4—C23—C24	109.58 (19)	C49—C50—H50A	116.4
N4—C23—H23A	109.8	C52—C51—C50	60.13 (19)
C24—C23—H23A	109.8	C52—C51—H51A	117.8
N4—C23—H23B	109.8	C50—C51—H51A	117.8
C24—C23—H23B	109.8	C52—C51—H51B	117.8
H23A—C23—H23B	108.2	C50—C51—H51B	117.8
C25—C24—C26	60.15 (19)	H51A—C51—H51B	114.9
C25—C24—C23	119.1 (3)	C51—C52—C50	59.88 (17)
C26—C24—C23	117.2 (2)	C51—C52—H52A	117.8
C25—C24—H24A	116.2	C50—C52—H52A	117.8
C26—C24—H24A	116.2	C51—C52—H52B	117.8
C23—C24—H24A	116.2	C50—C52—H52B	117.8
C24—C25—C26	60.62 (19)	H52A—C52—H52B	114.9
C24—C25—H25A	117.7	C8—N1—C12	111.33 (16)
C26—C25—H25A	117.7	C8—N1—C7	112.60 (17)
C24—C25—H25B	117.7	C12—N1—C7	113.60 (18)
C26—C25—H25B	117.7	C15—N2—C14	114.56 (18)
H25A—C25—H25B	114.8	C15—N3—C16	122.93 (17)
C25—C26—C24	59.23 (17)	C15—N3—C17	119.56 (16)
C25—C26—H26A	117.8	C16—N3—C17	117.39 (16)
C24—C26—H26A	117.8	C15—N4—C23	121.46 (19)
C25—C26—H26B	117.8	C15—N4—H4A	119.3
C24—C26—H26B	117.8	C23—N4—H4A	119.3
H26A—C26—H26B	115.0	C38—N5—C34	111.16 (17)
C32—C27—C28	119.6 (3)	C38—N5—C33	112.78 (17)
C32—C27—H27A	120.2	C34—N5—C33	114.17 (18)
C28—C27—H27A	120.2	C41—N6—C40	114.71 (18)
C27—C28—C29	120.1 (3)	C41—N7—C42	122.91 (17)
C27—C28—H28A	120.0	C41—N7—C43	118.94 (16)
C29—C28—H28A	120.0	C42—N7—C43	118.11 (16)
C28—C29—C30	120.8 (2)	C41—N8—C49	121.49 (18)
C28—C29—H29A	119.6	C41—N8—H8A	119.3
C30—C29—H29A	119.6	C49—N8—H8A	119.3
C31—C30—C29	118.0 (2)	C14—S1—C9	90.92 (10)
C31—C30—C33	120.7 (2)	C40—S2—C37	90.98 (10)
			
C6—C1—C2—C3	0.0 (4)	C40—C39—C42—N7	−3.9 (3)
C1—C2—C3—C4	−1.2 (4)	C36—C39—C42—N7	174.3 (2)
C2—C3—C4—C5	1.4 (3)	C48—C43—C44—C45	−2.0 (4)
C2—C3—C4—C7	−178.0 (2)	N7—C43—C44—C45	175.9 (2)
C3—C4—C5—C6	−0.4 (3)	C43—C44—C45—C46	1.0 (4)
C7—C4—C5—C6	179.0 (2)	C44—C45—C46—C47	0.9 (4)
C2—C1—C6—C5	1.0 (4)	C45—C46—C47—C48	−1.7 (4)
C4—C5—C6—C1	−0.8 (4)	C44—C43—C48—C47	1.2 (3)
C3—C4—C7—N1	92.6 (3)	N7—C43—C48—C47	−176.7 (2)
C5—C4—C7—N1	−86.8 (3)	C46—C47—C48—C43	0.7 (4)
N1—C8—C9—C10	−15.3 (3)	N8—C49—C50—C51	87.6 (3)
N1—C8—C9—S1	164.97 (15)	N8—C49—C50—C52	156.6 (2)
C8—C9—C10—C13	−179.88 (19)	C49—C50—C51—C52	106.6 (3)
S1—C9—C10—C13	−0.1 (2)	C49—C50—C52—C51	−109.1 (3)
C8—C9—C10—C11	−0.7 (3)	C9—C8—N1—C12	49.5 (2)
S1—C9—C10—C11	179.01 (16)	C9—C8—N1—C7	178.39 (18)
C9—C10—C11—C12	−16.1 (3)	C11—C12—N1—C8	−69.4 (2)
C13—C10—C11—C12	163.0 (2)	C11—C12—N1—C7	162.26 (17)
C10—C11—C12—N1	49.2 (2)	C4—C7—N1—C8	−65.5 (3)
C9—C10—C13—C14	0.1 (3)	C4—C7—N1—C12	62.2 (3)
C11—C10—C13—C14	−179.0 (2)	N4—C15—N2—C14	−179.0 (2)
C9—C10—C13—C16	176.8 (2)	N3—C15—N2—C14	0.4 (3)
C11—C10—C13—C16	−2.3 (4)	C13—C14—N2—C15	−2.3 (3)
C16—C13—C14—N2	1.7 (3)	S1—C14—N2—C15	176.37 (17)
C10—C13—C14—N2	178.8 (2)	N2—C15—N3—C16	2.1 (3)
C16—C13—C14—S1	−177.09 (15)	N4—C15—N3—C16	−178.5 (2)
C10—C13—C14—S1	0.0 (2)	N2—C15—N3—C17	−173.9 (2)
C14—C13—C16—O1	−179.8 (2)	N4—C15—N3—C17	5.4 (3)
C10—C13—C16—O1	3.6 (4)	O1—C16—N3—C15	178.0 (2)
C14—C13—C16—N3	0.8 (3)	C13—C16—N3—C15	−2.6 (3)
C10—C13—C16—N3	−175.8 (2)	O1—C16—N3—C17	−5.9 (3)
C22—C17—C18—C19	−1.3 (3)	C13—C16—N3—C17	173.52 (17)
N3—C17—C18—C19	177.6 (2)	C18—C17—N3—C15	−96.9 (2)
C17—C18—C19—C20	0.1 (4)	C22—C17—N3—C15	82.0 (3)
C18—C19—C20—C21	1.0 (4)	C18—C17—N3—C16	86.9 (2)
C19—C20—C21—C22	−0.9 (4)	C22—C17—N3—C16	−94.3 (2)
C20—C21—C22—C17	−0.3 (4)	N2—C15—N4—C23	−2.4 (4)
C18—C17—C22—C21	1.4 (4)	N3—C15—N4—C23	178.3 (2)
N3—C17—C22—C21	−177.5 (2)	C24—C23—N4—C15	−174.6 (2)
N4—C23—C24—C25	−89.8 (3)	C37—C38—N5—C34	−51.0 (2)
N4—C23—C24—C26	−159.1 (3)	C37—C38—N5—C33	179.33 (18)
C23—C24—C25—C26	−106.4 (3)	C35—C34—N5—C38	70.4 (2)
C23—C24—C26—C25	109.7 (3)	C35—C34—N5—C33	−160.66 (18)
C32—C27—C28—C29	−0.5 (4)	C30—C33—N5—C38	61.6 (3)
C27—C28—C29—C30	−0.6 (4)	C30—C33—N5—C34	−66.5 (3)
C28—C29—C30—C31	1.4 (3)	N8—C41—N6—C40	178.4 (2)
C28—C29—C30—C33	−178.8 (2)	N7—C41—N6—C40	−1.0 (3)
C29—C30—C31—C32	−1.2 (3)	C39—C40—N6—C41	2.2 (3)
C33—C30—C31—C32	179.0 (2)	S2—C40—N6—C41	−176.73 (17)
C30—C31—C32—C27	0.1 (4)	N6—C41—N7—C42	−2.8 (3)
C28—C27—C32—C31	0.7 (4)	N8—C41—N7—C42	177.73 (19)
C31—C30—C33—N5	−95.4 (3)	N6—C41—N7—C43	174.8 (2)
C29—C30—C33—N5	84.8 (3)	N8—C41—N7—C43	−4.6 (3)
N5—C34—C35—C36	−48.3 (2)	O2—C42—N7—C41	−176.15 (19)
C34—C35—C36—C37	14.1 (3)	C39—C42—N7—C41	5.1 (3)
C34—C35—C36—C39	−163.4 (2)	O2—C42—N7—C43	6.2 (3)
C39—C36—C37—C38	−180.0 (2)	C39—C42—N7—C43	−172.54 (17)
C35—C36—C37—C38	2.1 (3)	C44—C43—N7—C41	−74.8 (3)
C39—C36—C37—S2	0.7 (2)	C48—C43—N7—C41	103.1 (2)
C35—C36—C37—S2	−177.19 (17)	C44—C43—N7—C42	103.0 (2)
C36—C37—C38—N5	15.6 (3)	C48—C43—N7—C42	−79.1 (3)
S2—C37—C38—N5	−165.14 (16)	N6—C41—N8—C49	−1.5 (3)
C37—C36—C39—C40	−1.1 (3)	N7—C41—N8—C49	177.9 (2)
C35—C36—C39—C40	176.6 (2)	C50—C49—N8—C41	−175.5 (2)
C37—C36—C39—C42	−179.3 (2)	N2—C14—S1—C9	−178.96 (19)
C35—C36—C39—C42	−1.6 (4)	C13—C14—S1—C9	−0.09 (17)
C42—C39—C40—N6	0.4 (3)	C10—C9—S1—C14	0.14 (18)
C36—C39—C40—N6	−178.0 (2)	C8—C9—S1—C14	179.88 (19)
C42—C39—C40—S2	179.45 (15)	N6—C40—S2—C37	178.57 (19)
C36—C39—C40—S2	1.0 (2)	C39—C40—S2—C37	−0.52 (17)
C40—C39—C42—O2	177.5 (2)	C36—C37—S2—C40	−0.12 (18)
C36—C39—C42—O2	−4.3 (4)	C38—C37—S2—C40	−179.5 (2)
